# A practical approach for scaling up the alternative strategy for the elimination of lymphatic filariasis in *Loa loa* endemic countries - developing an action plan

**DOI:** 10.1186/s41256-017-0032-0

**Published:** 2017-05-01

**Authors:** Louise A. Kelly-Hope, Michelle C. Stanton, Honorat G. M. Zouré, Boniface E. Kinvi, Alexei Mikhailov, Afework Tekle, Jonathan D. King

**Affiliations:** 1 Department of Parasitology, Liverpool School of Tropical Medicine, Liverpool, UK; 2 African Programme for Onchocerciasis Control, Ouagadougou, Burkina Faso; 3 Communicable Disease Unit, World Health Organization, Regional Office for Africa, Brazzaville, Congo; 4 Department of Control of Neglected Tropical Diseases, World Health Organization, Geneva, Switzerland

## Abstract

**Background:**

Lymphatic filariasis (LF) is a vector-borne parasitic disease that is being targeted for elimination through mass drug administration (MDA). The co-distribution of *Loa loa* in Central Africa poses a significant barrier to the expansion of the MDA due to risk of severe adverse events (SAEs) associated with the drug ivermectin that is routinely used. National LF programmes are yet to significantly scale up in co-endemic areas and need a practical approach to make preliminary decisions based on the mapping status and potential treatment strategies.

**Methods:**

We reviewed relevant data available to WHO and in the literature for LF-*L. loa* endemic countries to develop a simple method to support the scale-up of MDA to eliminate LF.

**Results:**

A basic model for national LF programmes to work from at the administrative or implementation unit (IU) level has been developed for LF – *L. loa* co-endemic countries. The model includes five practical steps, which comprise the development of a national filarial database and a simple classification system to help determine the mapping status and most appropriate treatment strategy. Steps are colour-coded and linked to a general decision tree, which is also presented.

**Conclusions:**

This IU-level model is simple to follow and will help LF elimination programmes develop an action plan and scale up the implementation of alternative treatment strategies in *L. loa* co-endemic areas. The model could be further developed to incorporate the additional complexity of IUs where an intervention is required to eliminate onchocerciasis, particularly in hypo-endemic areas where ivermectin has not been used.

## Background

Lymphatic filariasis (LF) is a disabling parasitic disease transmitted by mosquitoes, and is endemic across tropical regions of the world. LF is currently being targeted for elimination coordinated through the Global Programme to Eliminate LF (GPELF), which is driven by two main goals including i) interrupting transmission with mass drug administration (MDA) of albendazole in combination with ivermectin or diethylcarbamazine citrate (DEC), and ii) morbidity management and disability prevention (MMDP) for those affected by clinical conditions [1]. The GPELF has made significant progress with 62 of 73 countries having implemented MDA, and 18 of these 62 countries no longer requiring MDA [2]. However, there have been challenges in starting and scaling up MDA in a number of sub-Saharan African countries due to the co-distribution of the filarial infection caused by *Loa loa* (also known as loiasis, tropical eye worm), and the potential risk of severe adverse events (SAEs) associated with the ingestion of ivermectin in individuals with high *L. Loa* microfilarial (Mf) loads [3–6]. Therefore, in LF and *L. loa* co-endemic areas alternative treatment strategies that exclude the use of ivermectin are required to ensure safe treatment, adequate coverage and operational impact.

In 2012 the World Health Organization (WHO) developed a provisional strategy for interrupting LF transmission in loiasis endemic countries [7], which also took into account the large scale community-directed treatment with ivermectin (CDTI) being implemented by the African Programme for Onchocerciasis Control (APOC), targeting areas with greater than 20% nodule prevalence [8, 9]. The LF strategy recommends that where *L. loa* infection is present and onchocerciasis (oncho) is non-endemic or hypo-endemic (defined as less than 20% of nodule prevalence), MDA should be implemented with biannual albendazole in combination with vector control. In LF endemic areas where oncho endemicity is defined as meso- or hyper-endemic, the strategy can also be used if CDTi has not been implemented [10]. The addition of vector control is considered important given that similar *Anopheles* mosquitoes transmit LF and malaria, and the malaria control programmes currently scaling up insecticide-treated mosquito net distribution in particular, could help to accelerate the reduction in LF transmission [11–13]. The efficacy of this alternative strategy has been demonstrated in ongoing operational research from Republic of Congo [14].

There is now a priority to move forward and roll out the most appropriate treatment strategies for LF elimination in *L. loa* endemic countries, taking the filarial co-endemicity, oncho - CDTi history and mapping status into account. The first and most fundamental step is to develop a basic framework or model for national LF programmes to work from and make decisions at the administrative or implementation level based on all available data.

## Methods

We reviewed all of the following data available to WHO and in the literature for LF – *L. loa* endemic countries:

1. Subnational endemicity status of LF and L. loa
2. Progress of MDA or CDTi
3. Reported use of bed nets
4. Subnational population estimates

We identified key indicators required to inform the elimination strategy decisions. Then developed a simple method to support national programmes review available data on these indicators and make suggestions of how to obtain missing data necessary to plan appropriate MDA strategy recommended by WHO for LF elimination.

This paper presents a five step practical model/approach to be used in LF – *L. loa* co-endemic countries, and includes the development of a national filarial database and a simple classification system to help determine the mapping status and most appropriate treatment strategy. A general decision tree is also presented.

## Results

### Scope

There are 10 LF endemic countries in West and Central Africa that are co-endemic with *L. Loa* which may require alternative treatment strategies to interrupt transmission. These countries are diverse in terms of *L. Loa* prevalence as determined by the rapid assessment procedure for loiasis (RAPLOA), as well as population size at risk [6, 15], and include Angola (est. pop 7.9 mil at risk), Cameroon (est. pop 8.3 mil at risk), Central Africa Republic (CAR; est. pop 2.8 mil at risk), Chad (est. pop 0.3 mil at risk), Congo (est. pop 1.4 mil at risk), Democratic Republic of Congo (DRC; est. pop 43.9 mil at risk), Equatorial Guinea (est. pop 0.42 mil at risk), Gabon (est. pop 0.2 mil at risk), Nigeria (est. pop 79.4 mil at risk), and South Sudan (est. pop 9.8 mil at risk).

The administrative unit which a country uses as the basis for implementing MDA is defined as the implementation unit (IU), and is usually the district or equivalent for LF elimination programmes [16]. Based on IU endemicity status available at the end of 2014, there were an estimated 1911 IUs across the ten countries, where the alternative strategy could be implemented. In order for national LF Programmes to determine which IUs require albendazole MDA and where additional assessments might be necessary across their country, the following five practical steps are recommended.

#### Step 1: build a filarial-information database

First, it is essential to develop a database with information on filarial endemicity and CDTi status for each IU in the country. The filarial data are related to the three main filarial diseases - LF, loiasis and oncho. Five countries have not initiated MDA for LF, but have implemented CDTi for oncho. The MDA status data are related to the standard implementation of ivermectin for oncho, namely CDTi, and if it has ever been initiated or completed for either LF or oncho. Data on the endemicity and MDA status need to be collated from both historical and current national mapping data, Ministry reports, APOC/WHO sources, and the scientific literature, and entered into a simple spreadsheet or data file.

#### Step 2: classify the endemicity and mapping status

**Fig. 1:**
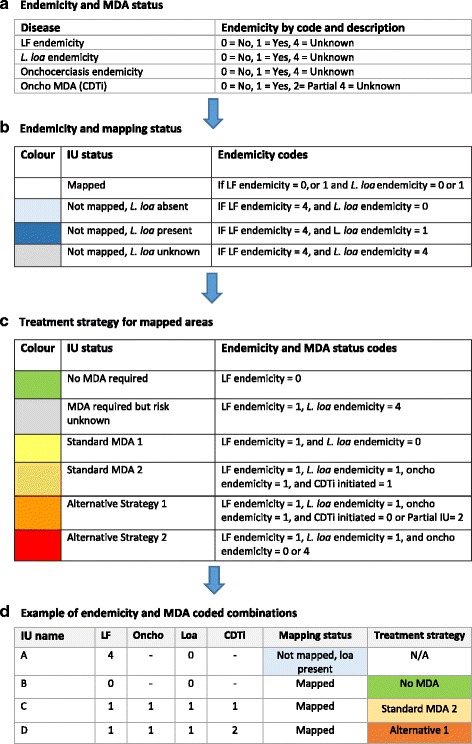
Tabulated filarial endemicity, MDA status, mapping status and treatment strategies. (a) Endemicity and MDA status (b) Endemicity and mapping status (c) Treatment strategy for mapped areas (d) Example of endemicity and MDA coded combinations

Second, a simple classification of the filarial endemicity is needed to determine whether any mapping needs to be conducted and what treatment strategies are recommended. For each IU, the data on LF, *L. loa* and oncho endemicity are classified and coded simply as non-endemic (No = 0), endemic (Yes = 1) or unknown (4 = Unknown). Examples of these coded data and how they help to determine the mapping status and treatment strategies are shown in Fig. 1a-d.

Where endemicity is unknown, new surveys may be needed. The combination of the LF and *L. loa* endemicity codes are used to define four mapping status categories as outlined in Fig. 1b. The first category includes i) ‘Mapped’ = if LF has been mapped and LF is endemic (code = 1) or non-endemic (code = 0), indicating that mapped endemic areas need to proceed and consider the most appropriate treatment strategies. Non-endemic areas require no treatment. The following three categories are related to unmapped areas and include ii) ‘Not Mapped, *L. loa* Absent ‘if the LF status is unknown (code = 4) and *L. loa* is non-endemic (code = 0), iii) ‘Not Mapped, *L. loa* Present’ = if LF is unknown (code = 4) and *L. loa* is endemic (code = 1), and iv) ‘Not Mapped, *L. loa* Unknown’ if the LF and *L. loa* endemicity indicators were both unknown (code = 4).

#### Step 3: determine the status of current/ongoing treatment strategies

Third, the status of MDA with ivermectin or CDTi for each IU must be determined. MDA has been initiated (code = 1) or not (code = 0) across the entire IU, or partially in selected areas. Communication with sub-national levels may be required to understand whether any preventive chemotherapy is ongoing in the IU. Because oncho transmission zones are not consistent with LF administrative/implementation units, CDTi may be implemented only in a part of an IU, i.e. villages in selected riverine areas instead of the entire IU. Therefore, the extent of CDTi within the IU should be noted i.e. IU/district-wide (No = 0, Yes = 1), or Partial IU areas (Partial = 2) or Unknown (Unknown = 4).

#### Step 4: define the required treatment strategy

Fourth, to determine the treatment strategy of an IU, the combination of the endemicity, mapping and MDA status codes are used to define six categories as outlined in Fig. 1c. The first two categories include i) ‘No MDA required’ = if LF is mapped and endemicity is non-endemic (code = 0), and ii) ‘MDA required but risk unknown’ if LF is endemic (code = 1), and *L. loa* is unknown (code = 4), indicating that *L. Loa* mapping may be required if *L. loa* could be endemic (reported cases, documented vector, or vector habitat). However, the alternative strategy of biannual albendazole in combination with vector control may start while the *L. loa* mapping is being conducted and the associated risk assessment completed.

The next two categories relate to standard MDA, and include iii) ‘Standard MDA 1’ = if LF endemicity is endemic (code = 1), and *L. loa* endemicity is non-endemic (code = 0) and iv) ‘Standard MDA 2’ if LF, *L. loa* and oncho are endemic (code = 1) and CDTi has been initiated in the IU (code = 1) in all sub-areas. Here, it is important to establish links with the national oncho programme to assess the treatment duration, and therapeutic and geographical coverage rates as low or limited coverage, and/or areas where CDTi is just starting, may have pockets for potential SAEs. Other factors such as the transience of communities, migration patterns and population turnover (i.e. birth/deaths rates) should also be considered.

The final categories relate to alternative treatments, and include v) ‘Alternative strategy 1’ if LF, *L. loa* and oncho are endemic (code = 1) and CDTi has not been initiated (code = 0) or has not been implemented in all sub-areas of the IU (code = 2), and vi) ‘Alternative Strategy 2’ if LF and *L. loa* are endemic (code = 1), and oncho is non-endemic (code = 0) or unknown (code = 4), and CDTi has not been initiated (code = 0). Here, is it important to establish links with the national malaria programme and assess vector control type, duration and geographical coverage as this may help to facilitate the scale up of these strategies.

#### Step 5: developing a decision tree and maps

**Fig. 2:**
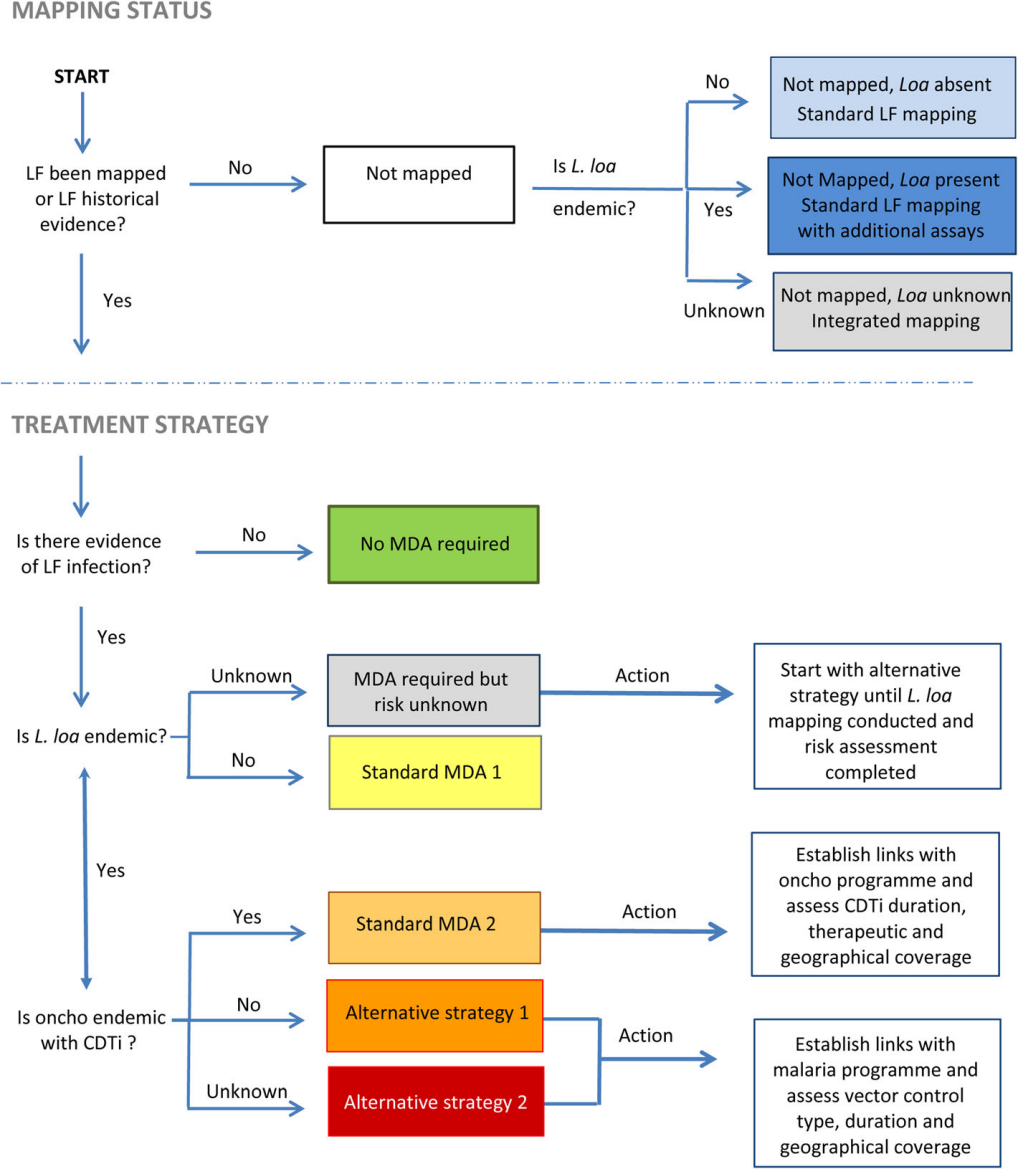
Decision tree to determine the mapping and/or treatment strategy process

Based on the above information, a simple decision tree can be developed for each country to inform the steps at IU–level as shown in Fig. 2. This decision tool supplements the filarial database and can be adapted to national programmatic policy.

For ‘Mapping Status’, the methodology will depend on the *L. loa* co-endemicity, and if ‘absent’ then standard LF mapping may be conducted, whereas if ‘present’ then additional assay/new diagnostic tools may be needed. If the *L. loa* status is ‘unknown’ and suspected to be present, then integrated mapping may be appropriate at different spatial scales using a combination of tools (e.g. BinaxNOW Filariasis immunochromatographic test (ICT) or Alere Filariasis Test Strip (FTS) Alere, Scarborough, ME, United States) [17], Mf and RAPLOA. For ‘Treatment Strategy’, the approach will depend on *L. loa* and oncho endemicity and CDTi status. IUs requiring more information before determining the strategy will be highlighted through this process. Links with oncho elimination and malaria programmes need to be developed to ascertain MDA status and vector control duration and coverage rates.

**Fig. 3:**
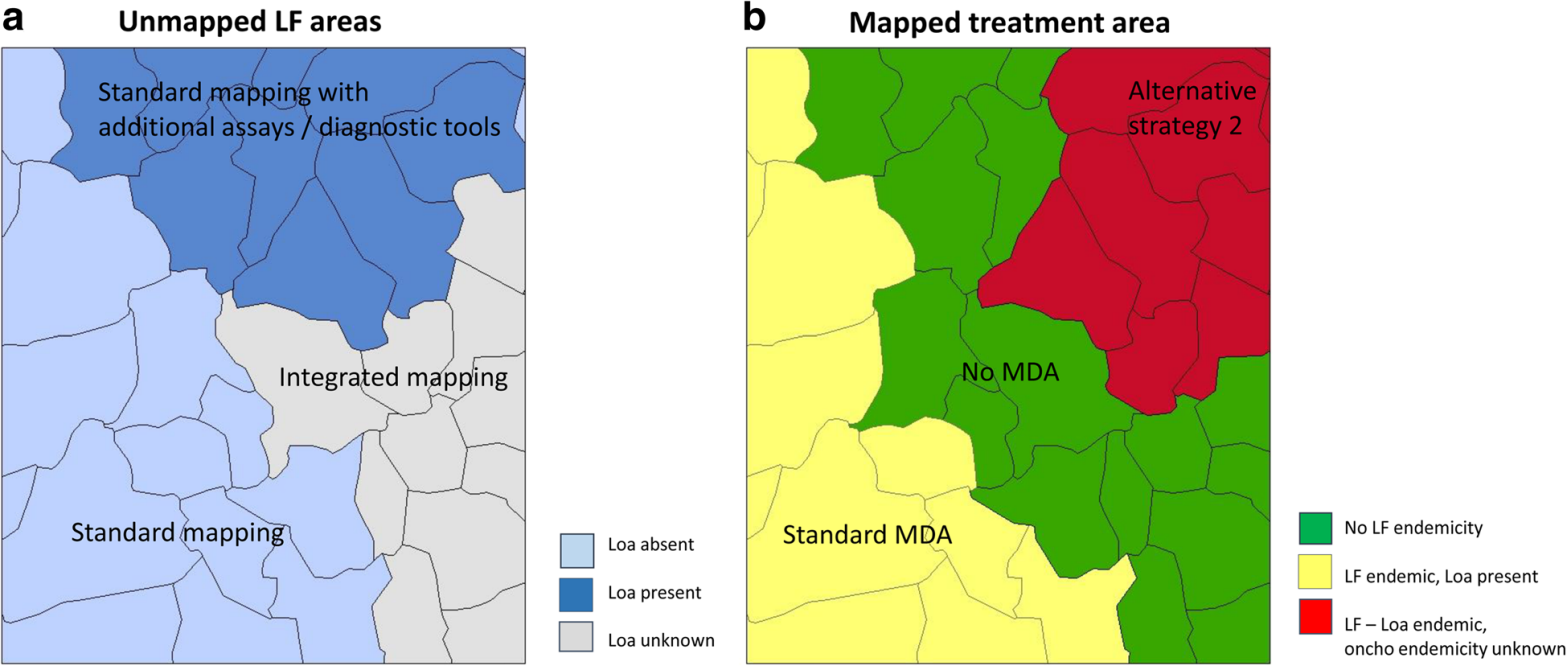
Example of maps highlighting mapping and treatment requirements in LF – Loa co-endemic area. (a) Unmapped LF areas (b) Mapped treatment areas

These data and categories can further be colour-coded to develop maps and help to highlight the geographical patterns of the mapping status of each IU, the most appropriate treatment strategies and where potential alternative mapping approaches can be considered. Figure 3a and b present examples of mapping status and treatment strategy maps within an area of a country, and highlight how the IUs may need different resources to confirm the LF endemicity and treatment within an IU.

## Discussion

These five practical steps are fundamental for LF – *L. loa* co-endemic country programmes to plan and scale up the implementation of biannual albendazole MDA. This initial model/approach is focussed on developing a national filarial database, reviewing the co-endemicity, mapping needs and treatment requirements at IU-level as a starting point. In principle, the scaling up of this readily available and safe alternative MDA strategy is possible without further detailed assessment of LF and *L. loa*. The required medicines are donated and available through WHO for requesting countries [18]. With the additional necessary commitment and resources for distribution, monitoring and evaluation, countries can get on track towards meeting the elimination target. However, it will be important to be aware of potential challenges related to the logistics of mobilising populations for twice yearly treatment and ensuring high bed net coverage. It is recommended that LF – *L. loa* co-endemic country programmes document and share the challenges as they arise, so that all programmes can benefit and learn from each other’s experience.

The recent reports of an association of high-density *L. loa* microfilaremia with ‘false’ positive ICT indicate a potential for overestimating LF endemicity [19, 20]. Further investigation of such an association is warranted. Any additional mapping, monitoring or transmission assessment surveys (TAS) for LF in loiasis endemic areas should include the collection of additional blood specimens for other filarial diagnostic assays [21]. New mapping approaches and tools might be required in known *L. loa* co-endemic areas. The new diagnostic Cell Scope Loa devise provides a tool with the capacity to quickly and affordably assess the *L. loa* positivity of individual patients, potentially allowing for ivermectin-based treatment to occur in a selective manner in LF - *L. loa* co-endemic areas and could be of critical importance in high risk SAE locations requiring alternative strategies [22, 23]. Further, in areas where LF and *L. loa* endemicity is suspected but unknown, an ‘integrated mapping’ method using a combination of FTS, MF for both LF and *L. loa*, and RAPLOA may help determine the extent and/or ecological pattern of co-endemicity, and provide further insights into the best diagnostic tools to use. A combination of micro- integrated- filarial mapping has previously been used to determine high *L. Loa* prevalence areas where ivermectin should not be used [24, 25]. The advantages and disadvantages of each mapping strategy should be carefully considered.

For this first step, we did not incorporate oncho elimination strategies. However, the same approach could be used for identifying the action required to define treatment strategies and help to develop a practical and safe action plan for oncho elimination. The Cell Scope Loa devise is probably more critical to use in oncho-*L. loa* endemic areas as there is currently no effective alternative strategy. Micro-integrated-filarial mapping may also be considered in the decision to expand ivermectin use in CDTi naive areas for elimination of oncho, especially in ‘hypo-endemic hotspots’ where oncho transmission is low, and the risk of *L. Loa* and SAEs is high [9, 26]. In addition to new mapping and diagnostic approaches, it is essential that LF Programmes now collaborate closely with the oncho and malaria programmes to collect and compile data on oncho CDTi and vector control therapeutic and geographical coverage rates for each IU. New links with soil transmitted helminths (STH) programmes will also be important as they scale up biannual albendazole MDA and potentially move into co-endemic areas [27]. Compiling this type of cross-programmatic data, and developing interlinking intervention databases and maps will be necessary to assess the geographical overlap, and determine if these different interventions have been - are - or will be - sufficient to provide impact and elimination potential for LF in a complex, dynamic environment [28].

## Conclusion

This paper presents a five step practical model/approach to be used in LF – *L. loa* co-endemic countries, and includes the development of a national filarial database and a simple classification system to help determine the mapping status and most appropriate treatment strategy. LF data templates for the ten countries have already been developed based on reported endemicity data or in literature to facilitate discussion and planning. This will help LF elimination programmes develop an action plan and start to scale up the implementation of treatment strategies. Further, the model could be further developed to incorporate the additional complexity of oncho, particularly in hypo-endemic areas.

## Abbreviations

APOC: African programme for onchocerciasis control
CAR: Central Africa Republic
CDTi: Community-directed treatment with ivermectin
DRC: Democratic Republic of Congo
DEC: Diethylcarbamazine citrate
GPELF: Global programme to eliminate lymphatic filariasis
ICT: Immunochromatographic test
IU: Implementation unit
FTS: Filariasis test strip
LF: Lymphatic filariasis
MDA: Mass drug administration
MF: Microfilaria
MMDP: Morbidity management and disability prevention
RAPLOA: Rapid assessment procedure for *Loa loa*
SAE: Severe adverse event
STH: Soil transmitted helminths
WHO: World Health Organization
